# A sodium calcium arsenate, NaCa(AsO_4_)

**DOI:** 10.1107/S160053681104428X

**Published:** 2011-11-02

**Authors:** Jinru Lin, Wei Sun, Jin-Xiao Mi, Yuanming Pan

**Affiliations:** a Department of Geological Sciences, University of Saskatchewan, Saskatoon, SK, Canada S7N 5E2; b Department of Materials Science and Engineering, College of Materials, Xiamen University, Xiamen 361005, Fujian Province, People’s Republic of China

## Abstract

The title compound, NaCa(AsO_4_), was synthesized using a hydro­thermal method at 633–643 K. It has a dense structure composed of alternating layers of distorted [CaO_6_] octa­hedra and layers of [AsO_4_] tetra­hedra and distorted [NaO_6_] octa­hedra, stacked along the *a* axis. The As, Ca and two O atoms lie on the mirror plane at *y* = 1/4 (*i.e.* 4*c*), while the Na atom lies on an inversion centre (1/2, 1/2, 0) (*i.e.* 4*b*). Each distorted [CaO_6_] octa­hedron shares four equatorial common O vertices with four neighboring octa­hedra, forming a layer parallel to (100), whereas each distorted [NaO_6_] octa­hedron shares two opposite edges with two neighboring ones, forming a chain running along [010]. Each isolated [AsO_4_] tetra­hedron shares two edges with two different [NaO_6_] octa­hedra in one [NaO_6_] chain and a vertex with another chain. Simultaneously the above [AsO_4_] tetra­hedron located in a four-membered [CaO_6_] ring shares one edge of its base facet with one [CaO_6_] octa­hedron and three corners with three other [CaO_6_] octa­hedra of one [CaO_6_] layer, and the remaining apex is shared with another [CaO_6_] layer. [NaO_6_] octa­hedra and [CaO_6_] octa­hedra are linked to each other by sharing edges and vertices.

## Related literature

For general background, see: Smedley & Kinniburgh (2002 ▶); Zhu *et al.* (2006 ▶); Rodríguez *et al.* (2008 ▶). For related structures, see: Graia *et al.* (1999 ▶) for CaNa_2_(AsO_4_)_3_; IJdo (1982) ▶ for NaCa(VO_4_); Ben Amara *et al.* (1983 ▶) for buchwaldite, NaCa(PO_4_); Bredig (1942) ▶ for NaCa(PO_4_).

## Experimental

### 

#### Crystal data

NaCa(AsO_4_)
*M* *_r_* = 201.99
Orthorhombic, 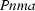

*a* = 11.486 (2) Å
*b* = 6.6615 (14) Å
*c* = 5.2406 (11) Å
*V* = 400.97 (15) Å^3^
*Z* = 4
Mo *K*α radiation
μ = 9.73 mm^−1^
*T* = 173 K
0.18 × 0.12 × 0.10 mm

#### Data collection

Bruker SMART CCD area-detector diffractometer
Absorption correction: multi-scan (*SADABS*; Bruker, 2001 ▶) *T*_min_ = 0.273, *T*_max_ = 0.443
2163 measured reflections
505 independent reflections
498 reflections with *I* > 2σ(*I*)
*R*_int_ = 0.035

#### Refinement

*R*[*F* ^2^ > 2σ(*F* ^2^)] = 0.021
*wR*(*F* ^2^) = 0.067
*S* = 1.03
505 reflections
42 parameters
Δρ_max_ = 0.83 e Å^−3^
Δρ_min_ = −0.74 e Å^−3^

### 

Data collection: *SMART* (Bruker, 2001 ▶); cell refinement: *SAINT* (Bruker, 2001 ▶); data reduction: *SAINT*; program(s) used to solve structure: *SHELXS97* (Sheldrick, 2008 ▶); program(s) used to refine structure: *SHELXL97* (Sheldrick, 2008 ▶); molecular graphics: *DIAMOND* (Brandenburg, 2011 ▶) and *ATOMS* (Dowty, 2004 ▶); software used to prepare material for publication: *SHELXL97*.

## Supplementary Material

Crystal structure: contains datablock(s) global, I. DOI: 10.1107/S160053681104428X/br2179sup1.cif

Structure factors: contains datablock(s) I. DOI: 10.1107/S160053681104428X/br2179Isup2.hkl

Additional supplementary materials:  crystallographic information; 3D view; checkCIF report

## Figures and Tables

**Fig. 1.**
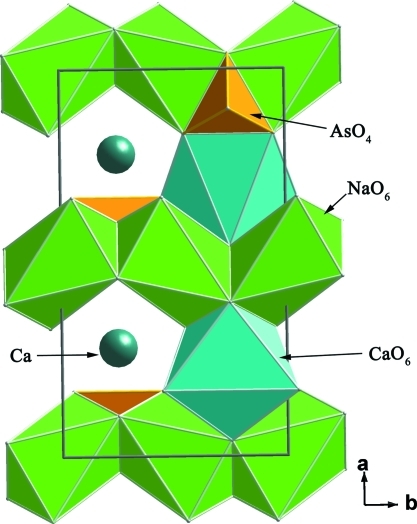
The crystal structure of NaCa(AsO4) plotted in projection along [001].

**Fig. 2.**
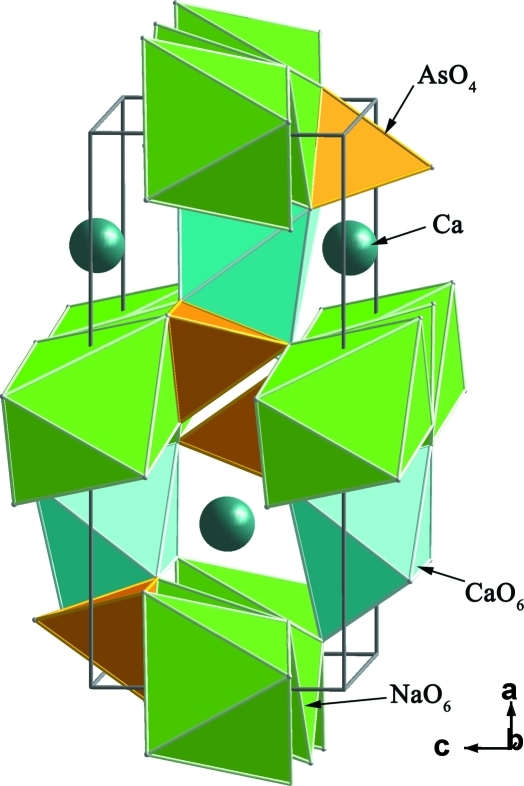
The crystal structure of NaCa(AsO4) plotted in projection along a direction near [010].

**Fig. 3.**
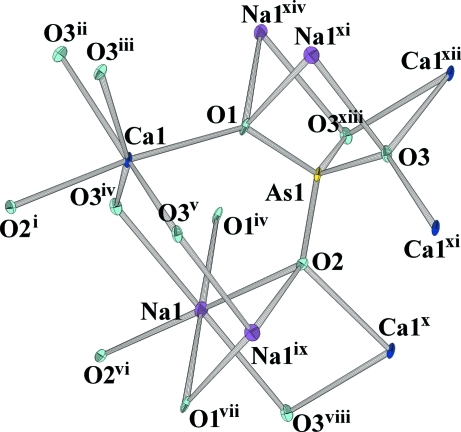
Coordination environment of Ca, Na, As and O atoms, with displacement ellipsoids drawn at the 50% probability level (symmetry codes: (i) -0.5+x, 0.5-y, 0.5-z; (ii) -0.5+x, 0.5-y, 1.5-z; (iii) -0.5+x, y, 1.5-z; (iv) 1-x, 0.5+y, 1-z; (v) 1-x, -y, 1-z; (vi) 1-x,0.5+y, -z; (vii) x, y, -1+z; (viii) x, 0.5-y,-1+z; (ix) 1-x, -0.5+y, -z; (x) 0.5+x, 0.5-y, 0.5-z; (xi) 1-x, -0.5+y, 1-z; (xii) 0.5+x, 0.5-y, 1.5-z; (xiii) x, 0.5-y, z; (xiv) x, y, z+1).

## supplementary crystallographic information

### Comment

Arsenic, a highly toxic pollutant in surface and ground waters, poses serious health and environmental problems all around the world (Smedley & Kinniburgh, 2002). One common method for immobilization and remediation of arsenic contamination in aqueous environments is through co-precipitation with calcium by forming various Ca-arsenate compounds (Zhu *et al.*, 2006). To date, more than 20 Ca-arsenate compounds have been reported, but only a few of them have their crystal structures determined (Rodríguez *et al.*, 2008). Knowledge about the crystal structures of Ca-arsenate compounds is important for better understanding their stabilities and potential applications for remediation of arsenic contamination in aqueous environments. Herein we report on the hydrothermal synthesis and crystal structure of a new compound NaCa(AsO_4_), which is the second sodium calcium arsenate after CaNa_2_(AsO_4_)_3_ (Graia *et al.*, 1999).

The crystal structure of the title compound differs from those of its phosphorus and vanadium counterparts (IJdo, 1982; Ben Amara *et al.*, 1983 & Bredig, 1942). The basic structural features of the title compound include [NaO_6_] octahedra, [AsO_4_] tetrahedra and [CaO_6_] octahedra. Sodium atoms are surrounded by six O-atoms forming distorted [NaO_6_] octahedra, which share edges to form chains running along [010] (Fig. 1–2). These octahedral chains are linked by isolated [AsO_4_] tetrahedra to form polyhedral sheets parallel to the (100) plane (Fig. 2). This linkage is made by each [AsO_4_] tetrahedron sharing edges with two [NaO_6_] octahedra in one chain and a vertex with another chain. These sheets are then linked together by [CaO_6_] octahedra *via* sharing edges and vertices(Fig. 1–3).

### Experimental

Single crystals of NaCa(AsO_4_) were synthesized using a hydrothermal method. A mixture of 0.5 mmol calcium nitrate Ca(NO_3_)_2_.H_2_O and 0.3 mmol sodium hydrogen arsenate heptahydrate Na_2_HAsO_4_.H_2_O was added to 5 ml of 2*M* NaOH solution. This mixed solution was then transferred into a 22 ml pressure vessel from Parr Instrument Company and heated to and maintained at a temperature of 633–643 K for 13 days. Solid products were filtered, washed with deionized water, and dried in the air. Crystals with a rectangular morphology were selected with a polarizing microscope for the collection of single-crystal X-ray diffraction data at 173 K.

### Crystal data

**Table:**

NaCa(AsO_4_)	*F*(000) = 384
*M**_r_* = 201.99	*D*_x_ = 3.346 Mg m^−^^3^
Orthorhombic, *P**n**m**a*	Mo *K*α radiation, λ = 0.71073 Å
Hall symbol: -P 2ac 2n	Cell parameters from 2163 reflections
*a* = 11.486 (2) Å	θ = 3.6–28.2°
*b* = 6.6615 (14) Å	µ = 9.73 mm^−^^1^
*c* = 5.2406 (11) Å	*T* = 173 K
*V* = 400.97 (15) Å^3^	Prism, colorless
*Z* = 4	0.18 × 0.12 × 0.10 mm

### Data collection

**Table:**

Bruker SMART CCD area-detector diffractometer	505 independent reflections
Radiation source: fine-focus sealed tube	498 reflections with *I* > 2σ(*I*)
graphite	*R*_int_ = 0.035
1200 images,Δω=1°, Exp time: 15 s. scans	θ_max_ = 28.2°, θ_min_ = 3.6°
Absorption correction: multi-scan (*SADABS*; Bruker, 2001)	*h* = −15→14
*T*_min_ = 0.273, *T*_max_ = 0.443	*k* = −5→8
2163 measured reflections	*l* = −6→6

### Refinement

**Table:**

Refinement on *F*^2^	Primary atom site location: structure-invariant direct methods
Least-squares matrix: full	Secondary atom site location: difference Fourier map
*R*[*F*^2^ > 2σ(*F*^2^)] = 0.021	*w* = 1/[σ^2^(*F*_o_^2^) + (0.0474*P*)^2^ + 0.5926*P*] where *P* = (*F*_o_^2^ + 2*F*_c_^2^)/3
*wR*(*F*^2^) = 0.067	(Δ/σ)_max_ = 0.001
*S* = 1.03	Δρ_max_ = 0.83 e Å^−^^3^
505 reflections	Δρ_min_ = −0.74 e Å^−^^3^
42 parameters	Extinction correction: *SHELXL97* (Sheldrick, 2008), Fc^*^=kFc[1+0.001xFc^2^λ^3^/sin(2θ)]^-1/4^
0 restraints	Extinction coefficient: 0.017 (2)

### Special details

**Table:**

Geometry. All e.s.d.'s (except the e.s.d. in the dihedral angle between two l.s. planes) are estimated using the full covariance matrix. The cell e.s.d.'s are taken into account individually in the estimation of e.s.d.'s in distances, angles and torsion angles; correlations between e.s.d.'s in cell parameters are only used when they are defined by crystal symmetry. An approximate (isotropic) treatment of cell e.s.d.'s is used for estimating e.s.d.'s involving l.s. planes.
Refinement. Refinement of *F*^2^ against ALL reflections. The weighted *R*-factor *wR* and goodness of fit *S* are based on *F*^2^, conventional *R*-factors *R* are based on *F*, with *F* set to zero for negative *F*^2^. The threshold expression of *F*^2^ > σ(*F*^2^) is used only for calculating *R*-factors(gt) *etc*. and is not relevant to the choice of reflections for refinement. *R*-factors based on *F*^2^ are statistically about twice as large as those based on *F*, and *R*- factors based on ALL data will be even larger.

### Fractional atomic coordinates and isotropic or equivalent isotropic displacement parameters (Å^2^)

**Table:**

	*x*	*y*	*z*	*U*_iso_*/*U*_eq_	
As1	0.59797 (3)	0.2500	0.56866 (7)	0.0046 (2)	
Na1	0.5000	0.5000	0.0000	0.0088 (3)	
Ca1	0.28059 (6)	0.2500	0.49344 (14)	0.0053 (2)	
O1	0.4611 (2)	0.2500	0.6849 (5)	0.0075 (5)	
O2	0.6038 (2)	0.2500	0.2482 (6)	0.0068 (6)	
O3	0.66769 (14)	0.0513 (3)	0.7038 (3)	0.0068 (4)	

### Atomic displacement parameters (Å^2^)

**Table:**

	*U*^11^	*U*^22^	*U*^33^	*U*^12^	*U*^13^	*U*^23^
As1	0.0014 (3)	0.0050 (3)	0.0073 (3)	0.000	0.00013 (11)	0.000
Na1	0.0076 (7)	0.0087 (7)	0.0101 (7)	0.0027 (6)	−0.0003 (6)	−0.0014 (6)
Ca1	0.0017 (4)	0.0051 (4)	0.0091 (4)	0.000	−0.0002 (2)	0.000
O1	0.0017 (10)	0.0096 (12)	0.0111 (12)	0.000	0.0023 (9)	0.000
O2	0.0042 (12)	0.0100 (13)	0.0062 (13)	0.000	0.0012 (8)	0.000
O3	0.0056 (8)	0.0048 (8)	0.0099 (8)	0.0014 (6)	−0.0024 (7)	0.0000 (7)

### Geometric parameters (Å, °)

**Table:**

As1—O2	1.681 (3)	Na1—O3^v^	2.4970 (18)
As1—O1	1.686 (2)	Na1—O3^vi^	2.4970 (18)
As1—O3^i^	1.7015 (17)	Ca1—O1	2.304 (3)
As1—O3	1.7015 (17)	Ca1—O3^vi^	2.3346 (19)
Na1—O1^ii^	2.3873 (19)	Ca1—O3^vii^	2.3346 (19)
Na1—O1^iii^	2.3874 (19)	Ca1—O2^viii^	2.393 (3)
Na1—O2	2.426 (2)	Ca1—O3^ix^	2.4393 (19)
Na1—O2^iv^	2.426 (2)	Ca1—O3^x^	2.4393 (19)
			
O2—As1—O1	113.48 (12)	O2^iv^—Na1—O3^vi^	81.96 (8)
O2—As1—O3^i^	113.41 (8)	O1—Ca1—O3^vi^	87.93 (6)
O1—As1—O3^i^	106.76 (8)	O1—Ca1—O3^vii^	87.93 (6)
O2—As1—O3	113.41 (8)	O3^vi^—Ca1—O3^vii^	118.57 (10)
O1—As1—O3	106.76 (8)	O1—Ca1—O2^viii^	173.87 (10)
O3^i^—As1—O3	102.14 (12)	O3^vi^—Ca1—O2^viii^	88.94 (6)
O1^ii^—Na1—O2	89.08 (7)	O3^vii^—Ca1—O2^viii^	88.94 (6)
O1^iii^—Na1—O2	90.92 (7)	O1—Ca1—O3^ix^	101.26 (7)
O1^ii^—Na1—O2^iv^	90.92 (7)	O3^vi^—Ca1—O3^ix^	87.52 (4)
O1^iii^—Na1—O2^iv^	89.08 (7)	O3^vii^—Ca1—O3^ix^	152.85 (7)
O1^ii^—Na1—O3^v^	67.60 (7)	O2^viii^—Ca1—O3^ix^	83.86 (7)
O1^iii^—Na1—O3^v^	112.40 (7)	O1—Ca1—O3^x^	101.26 (7)
O2—Na1—O3^v^	81.96 (8)	O3^vi^—Ca1—O3^x^	152.85 (7)
O2^iv^—Na1—O3^v^	98.04 (8)	O3^vii^—Ca1—O3^x^	87.52 (4)
O1^ii^—Na1—O3^vi^	112.40 (7)	O2^viii^—Ca1—O3^x^	83.86 (7)
O1^iii^—Na1—O3^vi^	67.60 (7)	O3^ix^—Ca1—O3^x^	65.72 (8)
O2—Na1—O3^vi^	98.04 (8)		

Symmetry codes: (i) *x*, −*y*+1/2, *z*; (ii) *x*, *y*, *z*−1; (iii) −*x*+1, −*y*+1, −*z*+1; (iv) −*x*+1, −*y*+1, −*z*; (v) *x*, −*y*+1/2, *z*−1; (vi) −*x*+1, *y*+1/2, −*z*+1; (vii) −*x*+1, −*y*, −*z*+1; (viii) *x*−1/2, *y*, −*z*+1/2; (ix) *x*−1/2, −*y*+1/2, −*z*+3/2; (x) *x*−1/2, *y*, −*z*+3/2.
